# Involvement of reactive oxygen species in endosperm cap weakening and embryo elongation growth during lettuce seed germination

**DOI:** 10.1093/jxb/eru167

**Published:** 2014-04-17

**Authors:** Yu Zhang, Bingxian Chen, Zhenjiang Xu, Zhaowan Shi, Shanli Chen, Xi Huang, Jianxun Chen, Xiaofeng Wang

**Affiliations:** Seed Science and Technology Lab, College of Life Sciences, South China Agricultural University, Guangzhou 510642, China

**Keywords:** Embryo elongation growth, endosperm cap weakening, hydrogen peroxide, lettuce, peroxidase, puncture force, reactive oxygen species, seed germination, sodium dichloroisocyanurate, superoxide radicals.

## Abstract

Endosperm cap (CAP) weakening and embryo elongation growth are prerequisites for the completion of lettuce seed germination. Although it has been proposed that the cell wall loosening underlying these processes results from an enzymatic mechanism, it is still unclear which enzymes are involved. Here it is shown that reactive oxygen species (ROS), which are non-enzymatic factors, may be involved in the two processes. In Guasihong lettuce seeds imbibed in water, O_2_·^–^ and H_2_O_2_ accumulated and peroxidase activity increased in the CAP, whereas its puncture force decreased. In addition, in the radicle, the increase in embryo growth potential was accompanied by accumulation of O_2_·^–^ and an increase in peroxidase activity. Imbibing seeds in 0.3% sodium dichloroisocyanurate (SDIC) reduced endosperm viability and the levels of O_2_·^–^, H_2_O_2_, and peroxidase activity in the CAP, whereas the decrease in its puncture force was inhibited. However, in the embryo, SDIC did not affect the accumulation of O_2_·^–^, peroxidase activity, and the embryo growth potential. As a result, SDIC caused atypical germination, in which the endosperm ruptured at the boundary between the CAP and lateral endosperm. ROS scavengers and ROS generation inhibitors inhibited the CAP weakening and also decreased the embryo growth potential, thus decreasing the percentage of seed germination. Exogenous ROS and ROS generation inducers increased the percentage of CAP rupture to some extent, and the addition of H_2_O_2_ to 0.3% SDIC enabled some seeds to undergo typical germination.

## Summary

Endosperm weakening and radicle elongation of lettuce seeds were separated by using sodium dichloroisocyanurate, and the roles of ROS in these processes were studied. A novel method was used for endosperm puncture force measurement.

## Introduction

Seed germination begins with water uptake and ends with the emergence of the radicle (RAD) through the surrounding seed tissues (Bewley *et al.*, 2013), and is a consequence of the competing interaction between the growth potential of the embryo and the limiting mechanical force of its surrounding tissues (Nambara *et al.*, 2010). Lettuce seed (actually a fruit, namely an achene with the testa fused to the pericarp), with its embryo enclosed completely in the seed coat and endosperm, is a good model in which to study the mechanisms of germination of these types of seed. The seed coat of mature lettuce seeds is a dead tissue which is broken immediately after imbibition, while the endosperm is composed of 2–3 layers of living cells and acts as the main barrier for the protrusion of the RAD (Jones, 1974). The rupture of the endosperm during seed germination is a result of two processes: growth of the embryo (normally the elongation of the RAD and/or hypocotyl), and weakening of the micropylar endosperm (or endosperm cap, CAP), by which its mechanical restraint is reduced. Although lettuce seed germination has been extensively studied, the mechanisms underlying the rupture of the CAP, especially its weakening process, are still largely unknown (Bewley, 1997; Dutta *et al.*, 1994; Nonogaki and Morohashi, 1999; Wang *et al.*, 2004).

It is widely accepted that during seed germination both the elongation of the RAD and the weakening of the CAP require cell wall loosening by wall hydrolases and/or transglycosylases, a process referred to as an enzymatic mechanism. In lettuce, the roles of numerous wall hydrolases, for example cellulase (EC 3.2.1.4) and some hemicellulose-degrading enzymes such as endo-β-mannanase (EC 3.2.1.78), α-galactosidase (EC 3.2.1.22), β-mannosidase (EC 3.2.1.25), and endo-β-xylanase (EC 3.2.1.8), in CAP weakening have been investigated (Bewley *et al.*, 1983; Bewley, 1997; Nonogaki and Morohashi, 1999; Wang *et al.*, 2004). However, almost all the results demonstrated that the activities of these enzymes do not increase prior to RAD protrusion, indicating that they are unlikely to play important roles. Hence, it is still unclear which enzymes are responsible for cell wall loosening in CAP weakening and RAD elongation growth in lettuce.

In addition to hydrolases and/or transglycosylases, non-enzymatic factors such as expansin and reactive oxygen species (ROS; superoxide radicals, hydrogen peroxide, and hydroxyl radicals) may also play roles in cell wall loosening. ·OH may directly cleave wall polysaccharides and thus loosen plant cell walls (Fry, 1998; Müller *et al.*, 2009; Schweikert *et al.*, 2000). The following mechanism for the formation of ·OH in the cell walls has been proposed (Chen and Schopfer, 1999; Müller *et al.*, 2007): NADPH oxidases on the plasma membrane catalyse the formation of apoplastic O_2_·^–^, which dismutates into H_2_O_2_ and O_2_. ·OH can be formed from O_2_·^–^ and H_2_O_2_ in the apoplast under catalysis by peroxidases (Schweikert et al., 2000, 2002). It is very difficult to detect ·OH due to its high reactivity and short life span (Müller *et al.*, 2009). However, the activity of peroxidases may reflect the production and accumulation of ·OH. Linkies *et al.* (2010) and Müller et al. (2007, 2009) proved that ROS, NADPH oxidases, and peroxidases contribute to CAP weakening and RAD elongation growth during seed germination in *Lepidium sativum*. Thus, it is of interest to know whether ROS also play a role in the two processes during lettuce seed germination.

Pavlišta and Haber (1970) reported a phenomenon of atypical germination in lettuce. They found that the RADs of lettuce seeds treated with sodium dichloroisocyanurate (SDIC) do not protrude from the tip of the micropylar endosperm, but instead from midway between the micropylar and cotyledonary ends. The embryos of some seeds expanded without RAD protrusion and resulted in embryo buckling in the endosperm. Thus, SDIC treatment provides an ideal model to investigate the mechanisms underlying CAP weakening and RAD elongation growth, since SDIC can separate the two processes.

The aim of the present study was to compare the patterns of ROS accumulation and related enzymes in the CAP and RAD of lettuce seeds imbibed in water and in 0.3% SDIC, and to see if ROS and related enzymes are involved in CAP weakening and RAD elongation growth.

## Materials and methods

### Plant materials and seed germination

Lettuce seeds (*Lactuca sativa* L. cv. Guasihong) were purchased from Guanghan Longsheng Seed Company, Sichuan province, China. Seeds were placed in Petri dishes (10cm in diameter) containing one layer of filter paper and 8ml of double-distilled water or 0.3% SDIC. The Petri dishes were incubated at 21 °C in continuous white light in a growth chamber (LRH-150-GB, Guangdong Medical Instrument Factory, Shaoguan, China). Endosperm rupture (i.e. seed germination) was scored using a Leica S6D stereomicroscope at 14, 16, 18, and 22h of imbibition in double-distilled water or at 18, 22, 28, and 34h of imbibition in 0.3% SDIC. Seeds were photographed using a Canon PowerShort A640 digital camera connected to the stereomicroscope.

### Incubation of excised embryos and endosperms

Seeds imbibed in double-distilled water or 0.3% SDIC for 2h were separated carefully into embryos and endosperms by using a scalpel and a forceps under a Leica S6D stereomicroscope. For this, seeds with the testa removed were cut slightly at the chalazal endosperm (without damage to the cotyledons) and pressed gently, then the embryo slid out of the endosperm. Subsequently, the excised embryos and endosperms were imbibed again in double-distilled water or 0.3% SDIC until harvest.

### TTC staining

Imbibed seeds or excised embryos and endosperms were stained in 0.5% triphenyltetrazolium chloride (TTC) at 30–35 °C for 0.5–1h. Images were taken as described above.

### Endosperm puncture force measurements

**Fig. 1.**
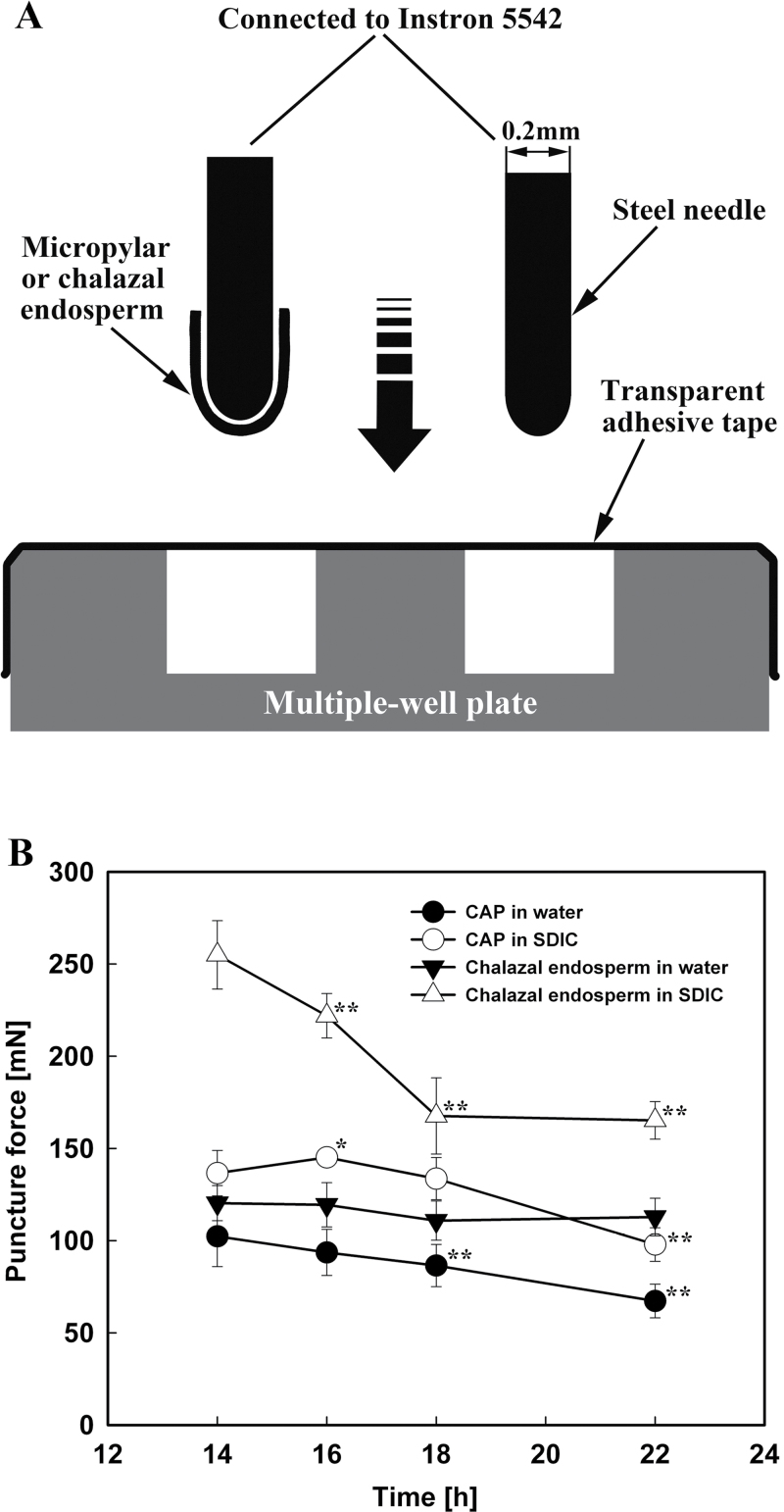
Endosperm puncture force measurements. (A) Schematic diagram of the measurement system with the main elements labelled. (B) Changes in the puncture force of the CAPs (circles) and chalazal endosperms (triangles) of lettuce seeds imbibed in water (filled symbols) or 0.3% SDIC (open symbols). Data are means ±SE of three biological replicates of 10 endosperms each. Significant differences in the data at 16, 18, and 22h from those at 14h of each treatment were assessed by Student’s *t*-test (*P<0.05, **P< 0.01).

A steel needle (0.2mm in diameter) with a round tip was used to puncture the micropylar or chalazal endosperm (Fig. 1A). This diameter was chosen because it is slightly smaller than that of the micropylar endosperms. After carefully inserting the round end into the inner space of the excised micropylar or chalazal endosperm, the other end of the needle was attached to an Instron 5542 electromechanical materials testing machine (Instron, USA). The needle with the micropylar or chalazal endosperm on it was moved downward at a speed of 10mm min^–1^ into one well of a multiple well plate, which was covered tightly and evenly by one layer of transparent adhesive tape, until the needle penetrated the tape. The force required to puncture both the endosperm and the tape was recorded as the maximum force from the force–displacement curves. To determine the puncture force of the adhesive tape, the needle (without any endosperm attached) was moved down through a well covered by tape. This was repeated 10 times, and the mean value was used (the measured values were very uniform; data not shown). The puncture force of the endosperm was calculated by subtracting the mean value of the puncture force for the tape alone from the measured values of the endosperm and tape combined. For the determination of endosperm puncture force, 30 micropylar or chalazal endosperms (three replications, with 10 endosperms in each) were used.

### Embryo growth potential (embryo mask area) measurements

**Fig. 2.**
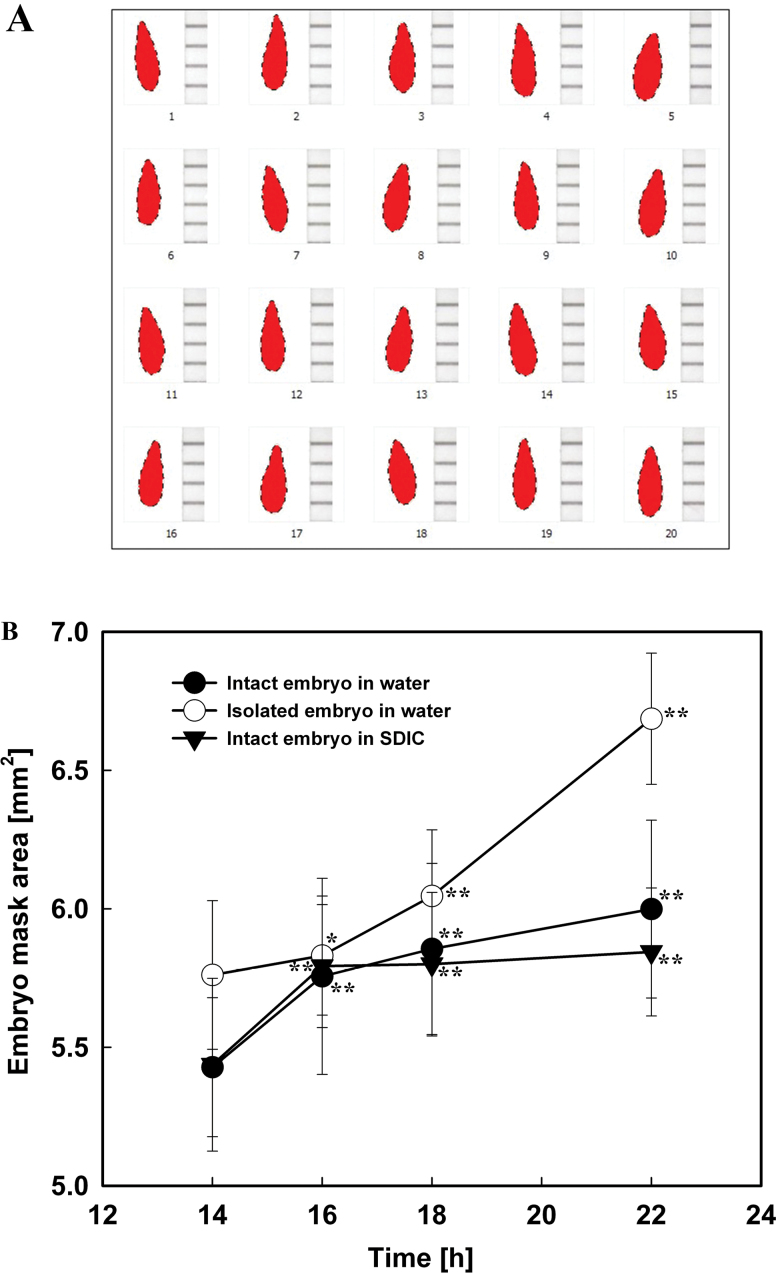
Embryo growth potential (embryo mask area) measurements. (A) Embryo masks converted from images as described in the text. (B) Changes in the mask area of intact (filled circles) and isolated (open circles) embryos of lettuce seeds imbibed in water, and intact embryos (filled triangles) imbibed in 0.3% SDIC. Data are means ±SE of three biological replicates of 20 seeds each. Significant differences in the data at 16, 18, and 22h from those at 14h of each treatment were assessed by Student’s *t*-test (*P<0.05, **P< 0.01). (This figure is available in colour at *JXB* online.)

Embryo mask areas were measured as described in Voegele *et al.* (2012), with minor modifications. Embryos from 60 seeds (three replications, with 20 seeds in each) imbibed in water or 0.3% SDIC at the indicated time points were carefully excised. The excised embryos were placed horizontally at the side of a paper ruler on a black ceramic plate and photographed with a binocular stereomicroscope (Leica S6D) connected to a digital camera. The background on each picture was deleted using Adobe Photoshop software, so that only the image of the embryo remained. The resulting images were converted to embryo mask areas as shown in Fig. 2A, using the image analysis software DUSP V1.0 T20120612-X1143 (Guangzhou Station for DUS Testing Center of New Plant Varieties, Ministry of Agriculture, PR China). The embryo mask areas calculated by this software were used to quantify the embryo growth potential.

### Histochemical localization and quantification of superoxide radicals

For histochemical localization of O_2_·^–^, NBT (nitroblue tetrazolium) staining was used as described in Oracz *et al.* (2012). Seeds with the testa removed, RADs, or CAPs were incubated in 1mM NBT in 10mM TRIS-HCl buffer (pH 7) at room temperature for 30, 5, and 30min respectively, then washed in the buffer and photographed. To quantify O_2_·^–^, the production rate (nmol O_2_·^–^ min^–1^ g FW^–1^) was analysed as described in Wang and Luo (1990). For this, 300 RADs or CAPs at the indicated imbibition time points were extracted in 2ml of 50mM phosphate buffer containing 1mM EDTA, 0.3% Triton X-100, and 2% PVP (pH 7.8). The homogenate was centrifuged at 12 000rpm for 20min. A 1ml aliquot of supernatant solution was mixed with 1ml of 50mM phosphate buffer (pH 7.8) and 1ml of 1mM hydroxylamine hydrochloride, and incubated at 25 °C for 1h. After the addition of 1ml of 17mM *p*-aminobenzenesulphonic acid and 1ml of 7mM α-naphthylamine, the mixture was incubated at 25 °C for 20min. The absorbance of the obtained solution was read at 530nm. A standard response curve was prepared with a known concentration of NO_2_^–^ using the same method as described above. Mean values ±SE of three biological replicates were calculated.

### Histochemical localization and quantification of hydrogen peroxide

Histochemical localization of H_2_O_2_ was carried out by DAB (3,3-diaminobenzidine) staining as described in Thordal-Christensen *et al.* (1997). Seeds with testa removed, RADs, or CAPs were incubated in 1mg ml^–1^ DAB-HCl (pH 3.8) at room temperature for 30min, then washed in water and photographed. H_2_O_2_ content (μmol g FW^–1^) was colorimetrically measured as described in Huang *et al.* (2011) with minor modifications. H_2_O_2_ was extracted by the homogenization of 300 RADs or CAPs at the indicated imbibition time points in 3ml of cold acetone. The homogenate was centrifuged at 12 000rpm for 15min. A 1ml aliquot of supernatant solution was mixed with 0.1ml of 5% titanium sulphate in concentrated HCl followed by the addition of 0.2ml of aqueous NH_3_ (25%) to precipitate the peroxide–titanium complex. The mixture was then centrifuged at 12 000rpm for 15min. The precipitate was solubilized in 3ml of 2mM H_2_SO_4_. The absorbance of the obtained solution was read at 415nm. A standard response curve was prepared with a known concentration of H_2_O_2_ using the same method as described above. Mean values ±SE of three biological replicates were calculated.

### Histochemical detection of peroxidase activity

Peroxidase activity was detected histochemically by TMB (3,3′,5,5′-tetramethylbenzidine) staining as described in Linkies *et al.* (2010). Seeds with the testa removed, RADs, or CAPs were incubated in 0.2% (w/v) TMB and 1mM H_2_O_2_ in 20mM phosphate buffer (pH 6.5) at room temperature for 30, 5, and 30min, respectively, then washed in the buffer and photographed.

### Seed treatments with ROS scavengers and ROS generation inhibitors

To investigate the effects of ROS scavengers on lettuce seed germination, 3mM CuCl_2_ or 30mM Tiron (scavengers for O_2_·^–^), 30mM KI or 50mM sodium pyruvate (scavengers for H_2_O_2_), and 10mM sodium benzoate or 30mM adenine (scavengers for ·OH) were used (Liszkay *et al.*, 2004).

Inhibitors of ROS generation, including 50 μM DPI (diphenylene iodonium chloride) or 10mM ZnCl_2_ (inhibitors for NADPH oxidases, the key enzyme for the generation of O_2_·^–^) and 0.3mM salicylic hydroxamate or 10mM methimazole (inhibitors for peroxidase activity, the key enzyme for the production of ·OH) were used (Kawano and Muto, 2000; Liszkay *et al.*, 2004; Müller *et al.*, 2009).

### Seed treatments with exogenous ROS and promoters of ROS generation

Solutions of 20 μM paraquat, 5mM H_2_O_2_, 20 μM NADPH, 5mM H_2_O_2_+20 μM Fe^2+^, and 5mM H_2_O_2_+20 μM Fe^2+^+1mM ascorbate were used as exogenous ROS or promoters of ROS generation in the imbibition medium (Fry, 1998; Müller *et al.*, 2009).

### Statistical tests

Student’s *t*-tests were conducted using the SAS software. All sample sizes and significance thresholds are indicated in the figure legends.

## Results

### Endosperm rupture occurred at the tip of the micropylar endosperm when imbibed in water, but at the connection region of the micropylar and lateral endosperm when imbibed in 0.3% SDIC

**Fig. 3.**
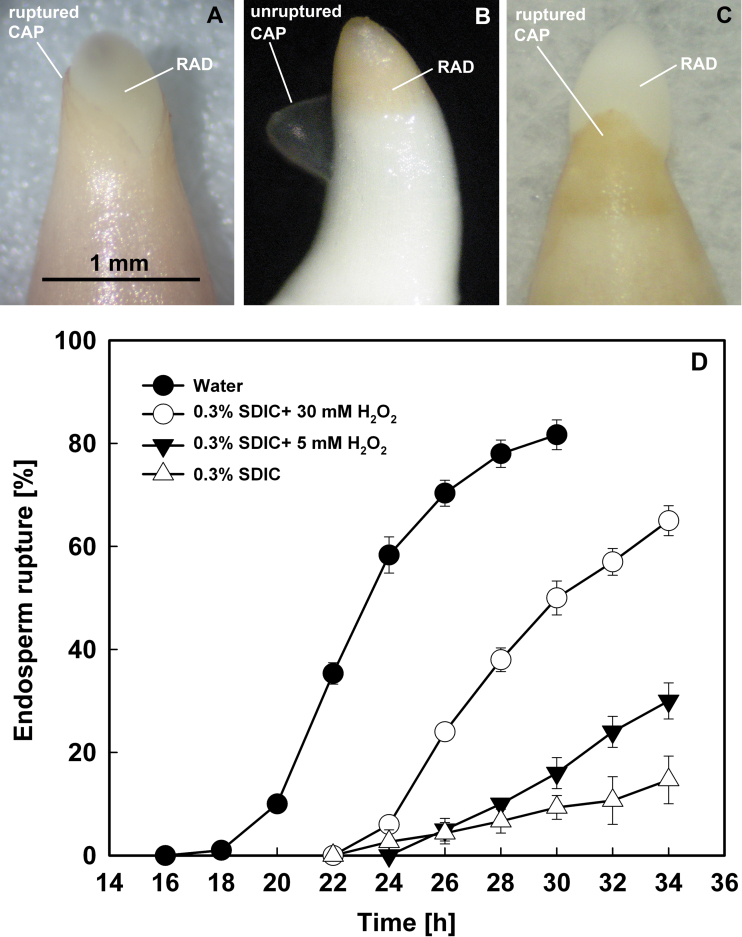
Typical (CAP ruptured) and atypical (endosperm ruptured at the region between the CAP and lateral endosperm) germination of lettuce seeds. (A) Typical germination in water. Scale bar=1mm. (B) Atypical germination in 0.3% SDIC. (C) Typical germination in 0.3% SDIC+5–10mM H_2_O_2_. (D) Germination time course. Data are means ±SE of three biological replicates of 100 seeds each. CAP, endosperm cap; RAD, radicle. (This figure is available in colour at *JXB* online.)

The endosperm of lettuce (cv. Guasihong) seeds began to rupture (first emergence of the RAD) at 18h of imbibition in water at 21 °C in continuous white light (Fig. 3D). The endosperm ruptured at the very tip of the CAP (Fig. 3A), which is defined as typical germination. The percentage of CAP rupture (i.e. completion of germination) increased gradually after 18h of imbibition and reached 80% at 30h.

However, when seeds were imbibed in 0.3% SDIC, the endosperm ruptured at the connecting region between the lateral and micropylar endosperm, and the CAP was pushed off intact by the expanding RAD (Fig. 3B), a phenomenon called atypical germination. Atypical germination started to occur from 22h of imbibition in 0.3% SDIC and increased gradually to ~20% by 34h of imbibition (Fig. 3D).

### CAP puncture force decreased when seeds were imbibed in water, but this process was inhibited by 0.3% SDIC

Using the biomechanical method described in the Materials and methods, the puncture force of the CAP and chalazal endosperm of the Guasihong lettuce seeds was measured (Fig. 1B). When seeds were imbibed in water for 14–18h, the puncture force of the CAP decreased gradually, and slightly faster after 18h, the time point at which the first seeds complete their germination (Fig. 3D). The puncture force at 18h, ~86 mN, appears to be a threshold value for the rupture of the CAP. The puncture force of the chalazal endosperm was higher than that of the CAP and remained almost constant during imbibition.

The puncture force of excised endosperms incubated in water was also measured, and it was found that the puncture force of excised CAPs was higher than that of intact CAPs, and a decrease also occurred in excised CAPs during imbibition, similar to intact endosperms (data not shown).

However, when seeds were imbibed in 0.3% SDIC, the puncture force of the endosperm (especially the chalazal endosperm) was significantly elevated (Fig. 1B). The reason for this is unclear. Although the puncture force of the CAP increased slightly at first (14–16h) and then decreased (16–22h) in 0.3% SDIC, the lowest value at 22h, ~98 mN, was still higher than that of the CAP at 18h of imbibition in water (86 mN). As stated above, the puncture force at 18h of imbibition in water is probably a threshold value for the rupture of endosperm. Hence, as the puncture force of the CAP was always higher than this threshold, the cap would not rupture during imbibition in 0.3% SDIC.

### Embryo growth potential (embryo mask area) increased when seeds were imbibed in both water and 0.3% SDIC

Measurement of the embryo mask area was used to quantify the changes in embryo growth potential. It was found that the embryo mask area increased gradually during imbibition in water, with the fastest speed at 14–16h (Fig. 2B). Seeds imbibed in 0.3% SDIC showed an increase in embryo mask area similar to that of the seeds imbibed in water. In addition, the embryo mask area values at each time point were also similar, except that at 22h the value in 0.3% SDIC was slightly lower than that in water. These results demonstrated that 0.3% SDIC did not inhibit embryo elongation growth in intact seeds.

The mask area of excised embryos incubated in water also increased, and was much higher than that of intact embryos enclosed in the endosperm at all time points except 16h (Fig. 2B), indicating that the endosperm limits the embryo growth. However, when the excised embryos were directly incubated in 0.3% SDIC, they stopped growing and became pale in colour (data not shown). It was reasoned that the excised embryos were damaged by direct contact with SDIC and lost viability, while the intact embryos could keep growing in 0.3% SDIC due to the protection of the enclosing endosperm. Therefore, the influence of SDIC on the viability of the endosperm and embryo of seeds was next analysed by TTC staining.

### CAP viability decreased after SDIC treatment

**Fig. 4.**
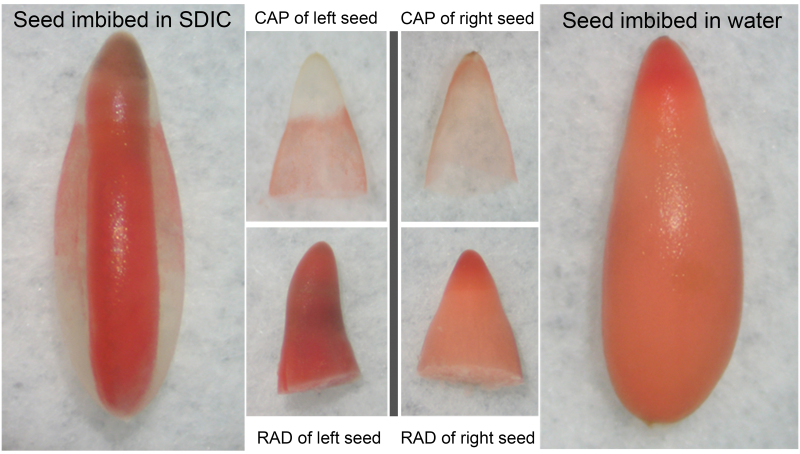
TTC staining of lettuce seeds imbibed in 0.3% SDIC or water. After staining, a whole seed was photographed and then the CAP and RAD were separated and photographed again. (This figure is available in colour at *JXB* online.)

To determine whether viability of the endosperm and embryo is influenced by SDIC, seeds imbibed in water or 0.3% SDIC were stained by TTC and photographed (Fig. 4). When imbibed in water, the embryo, CAP, and lateral endosperm were all stained. However, when imbibed in 0.3% SDIC, the CAP was the only tissue that was not stained. This demonstrated that SDIC specifically reduced the viability of the CAP, but not of the embryo and lateral endosperm.

### O_2_·^–^ was produced and accumulated in both the CAP and RAD of seeds imbibed in water, but this process in the CAP was inhibited by 0.3% SDIC

The production and accumulation of O_2_·^–^ were investigated by histochemical NBT staining in the CAP and RAD of lettuce seeds imbibed in water or 0.3% SDIC.

The experiment was first attempted using whole imbibed seeds, but the NBT staining failed. This might be caused by O_2_·^–^ not being produced during imbibition or by NBT not penetrating the endosperm. Imbibed seeds were then separated into endosperm and embryo before NBT staining and it was found that both endosperm and embryo were stained. This showed clearly that NBT cannot penetrate the endosperm layer. In addition, O_2_·^–^ must be produced at the inner side of the endosperm, because if it was produced and accumulated at the outer side, the intact seeds should have been stained. Therefore, seeds were dissected into endosperm and embryo before NBT staining in the following experiments.

**Fig. 5.**
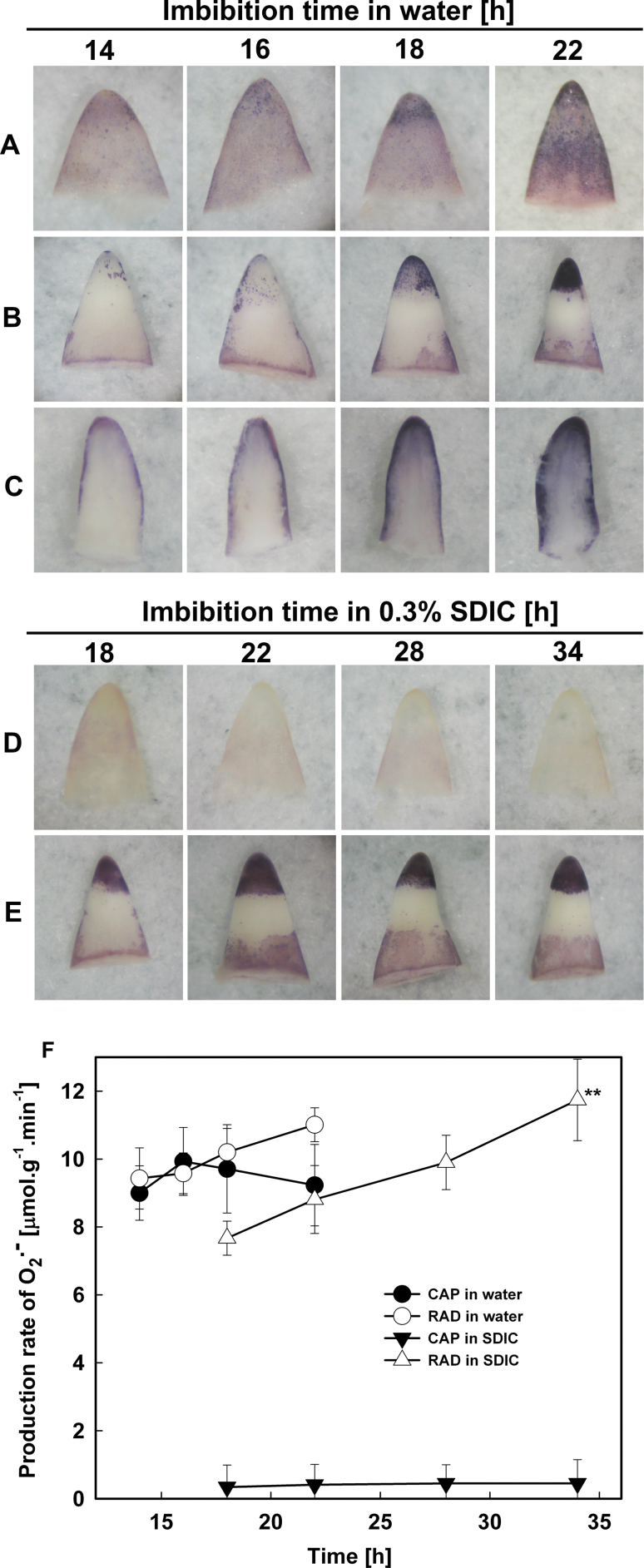
Histochemical staining by NBT (A–E) and determination of the production rate (F) of superoxide radicals in lettuce seeds imbibed in water (A–C) or 0.3% SDIC (D, E). (A, D) CAPs. (B, E) RADs. (C) Longitudinal section of RADs. Data are means ±SE of three biological replicates of 300 CAPs or RADs each. Significant differences in the data at 16, 18, and 22h from those at 14h of treatment in water, and at 22, 28, and 34h from those at 18h of treatment in 0.3% SDIC were assessed by Student’s *t*-test (*P<0.05, **P< 0.01). (This figure is available in colour at *JXB* online.)

When seeds were imbibed in water, the endosperm was stained by NBT and showed stronger staining at the CAP tip (Fig. 5A). The intensity of the staining increased with the imbibition time and was more evident after 18h, the time point at which the first seeds completed their germination (Fig. 3D). The intensity of the staining in the RAD (especially at the tip) also increased with the extension of imbibition in water, and was also more evident after 18h (Fig. 5B). To localize further the site of O_2_·^–^ accumulation inside the RAD, the stained (a somewhat longer staining time was required for the penetration of NBT) RAD was longitudinally sectioned and photographed (Fig. 5C). This showed that O_2_·^–^ was first detected in the epidermis and outer cortex, and in the vascular tissues after 18h of imbibition.

However, when seeds were imbibed in 0.3% SDIC, the intensity of the staining in the endosperm was reduced, especially at the CAP tip, which was almost not stained (Fig. 5D). However, the pattern of the staining in the RAD in 0.3% SDIC (Fig. 5E) was almost the same as that in water (compare the 18h and 22h time points in water and 0.3% SDIC). These results indicated that SDIC significantly inhibited the accumulation of O_2_·^–^ in the CAP, but not in the RAD.

The generation rate of O_2_·^–^ in the RAD and CAP was also quantified by colorimetry (Fig. 5F). The generation rate in the CAP increased before 16h of imbibition in water, and decreased thereafter, but remained at high levels, whereas the generation rate in the RAD continued to increase. When seeds were imbibed in 0.3% SDIC, however, the generation rate of O_2_·^–^ was very low in the CAP, whereas in the RAD it was still at relatively high levels and kept increasing during imbibition. The results of O_2_·^–^ quantification were consistent with that of histochemical staining.

### H_2_O_2_ was produced and accumulated only in the CAP of seeds imbibed in water, but was inhibited by 0.3% SDIC

The production and accumulation of H_2_O_2_ were investigated by performing DAB staining, and it was found that only the endosperm was stained in whole imbibed seeds and in isolated endosperms, suggesting that unlike NBT, DAB can penetrate the endosperm layer, or that H_2_O_2_ is produced on both sides (the outer and inner side) of the endosperm.

**Fig. 6.**
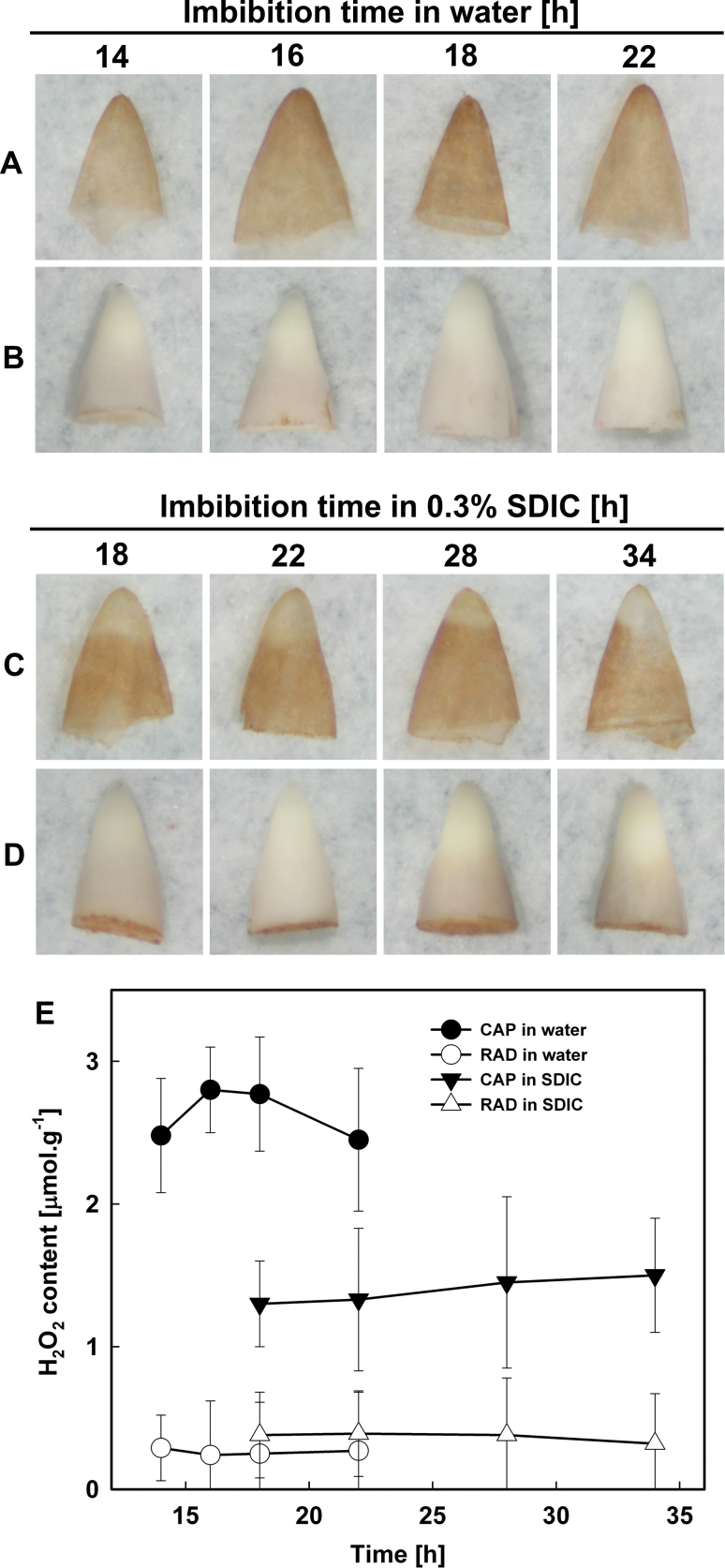
Histochemical staining by DAB (A–D) and quantification (E) of hydrogen peroxide in lettuce seeds imbibed in water (A, B) or 0.3% SDIC (C, D). (A, C) CAPs. (B, D) RADs. Data are means ±SE of three biological replicates of 300 CAPs or RADs each. Significant differences in the data at 16, 18, and 22h from those at 14h of treatment in water, and at 22, 28, and 34h from those at 18h of treatment in 0.3% SDIC were assessed by Student’s *t*-test (*P<0.05, **P< 0.01). (This figure is available in colour at *JXB* online.)

When seeds were imbibed in water, the endosperm was stained by DAB, with the strongest intensity at the CAP tip (Fig. 6A). The intensity of the staining increased during 14–18h of imbibition but decreased slightly thereafter, whereas the RAD was not stained at all during the whole imbibition time tested (Fig. 6B).

However, when seeds were imbibed in 0.3% SDIC, the intensity of the staining in the endosperm was reduced, especially in the CAP (Fig. 6C). Like imbibition in water (Fig. 6B), the embryo of seeds imbibed in 0.3% SDIC were not stained at all during the imbibition time period examined (Fig. 6D).

The content of H_2_O_2_ in the RAD and CAP was further quantified by colorimetry (Fig. 6E). The results showed that when seeds were imbibed in water, the H_2_O_2_ content in the CAP increased prior to the rupture of the CAP (18h of imbibition) and decreased after that, whereas H_2_O_2_ content in the RAD was very low. However, when seeds were imbibed in 0.3% SDIC, the H_2_O_2_ content in the CAP was greatly reduced, and that in the RAD was also very low, similar to imbibition in water. These results were in agreement with the histochemical staining.

### Peroxidase activity increased in both the CAP and RAD of seeds imbibed in water, but that in the CAP was inhibited by 0.3% SDIC

**Fig. 7.**
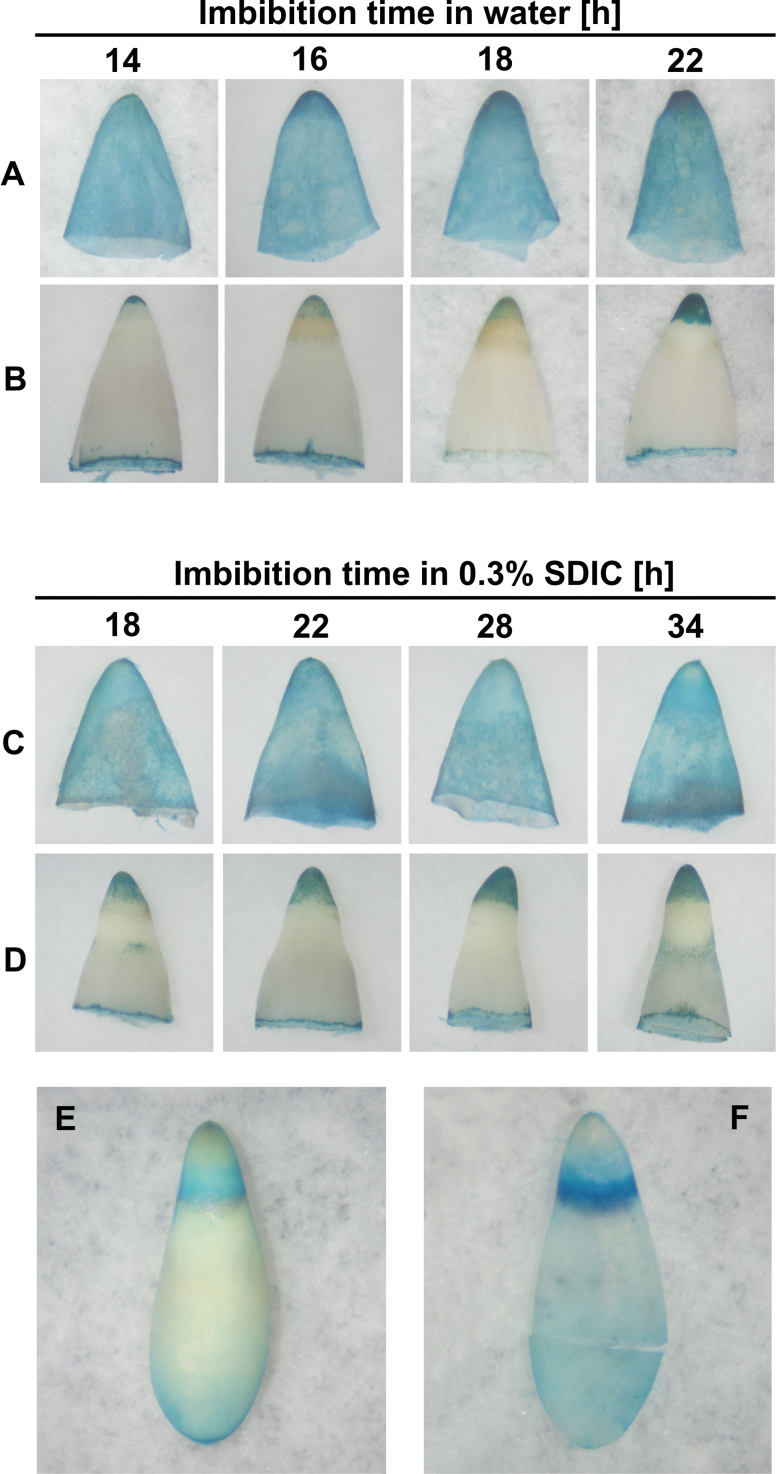
Histochemical detection of peroxidase activity by TMB staining in lettuce seeds imbibed in water (A, B) or 0.3% SDIC (C–F). (A, C) CAPs. (B, D) RADs. (E) Seed imbibed in 0.3% SDIC for 34h. (F) Endosperm separated from E. (This figure is available in colour at *JXB* online.)

Peroxidase activity in the CAP and RAD of lettuce seeds was localized by TMB staining. Whole imbibed seeds could be stained. However, after the stained seeds were dissected into embryo and endosperm, it was found that the colour was merely from the endosperm, while the embryo was not stained. To assess if the endosperm prevented the penetration of TMB and its direct contact with the embryo, the imbibed seeds were dissected into embryo and endosperm before TMB staining, and it was found that both the endosperm and RAD tip were stained (Fig. 7). This clearly indicated that the endosperm prevents the penetration of TMB when the whole seeds were used for staining. In addition, the staining was evenly distributed in the endosperm. However, when the CAP was turned inside out before staining, it was found that the staining in the tip of the CAP was more intense (Fig. 7A), indicating that the CAP tip had the highest peroxidase activity.

Using the method above, the changes in peroxidase activity were analysed in the CAP and RAD of seeds imbibed in water. The intensity of TMB staining in the tip of both the CAP and RAD increased with the extension of imbibition time (Fig. 7A, B). When seeds were imbibed in 0.3% SDIC, the intensity of TMB staining in the endosperm (especially in the CAP) was decreased (Fig. 7C), indicating that SDIC specifically reduced the peroxidase activity in the CAP. However, imbibition with SDIC did not change the TMB staining in the RAD (Fig. 7D), showing that SDIC did not influence the peroxidase activity in the embryo.

Interestingly, the connecting region between the CAP and lateral endosperm of some seeds imbibed in 0.3% SDIC was strongly stained, with a semi-circular appearance (Fig. 7E, F). This is the region where the endosperm ruptured when atypical germination occurred (Fig. 3B), implying that peroxidase activity is also involved in the endosperm rupture in atypical germination.

### CAP weakening and RAD elongation growth were inhibited by ROS scavengers and inhibitors of ROS generation

To investigate further the relationship between ROS and CAP weakening and RAD elongation growth, seeds were treated with ROS scavengers and inhibitors of ROS generation, and their effects on the puncture force of the CAP, the embryo growth potential, and the percentage of endosperm rupture were analysed (Table 1). The results showed that all ROS scavengers and ROS generation inhibitors tested greatly decreased the percentage of ruptured CAPs, indicating that ROS are important factors in the germination of lettuce seeds.

**Table 1.** Effect of ROS scavengers and inhibitors of ROS generation on the percentage of ruptured endosperm cap (CAP) of lettuce seeds

Treatments	CAP rupture (%)
H_2_O (control)	80±7
ROS scavengers	
O_2_·^–^ scavengers	
3mM CuCl_2_	10±5
30mM Tiron	35±6
H_2_O_2_ scavengers	
30mM KI	58±9
50mM sodium pyruvate	43±6
·OH scavengers	
10mM sodium benzoate	20±6
30mM adenine	6±3
ROS generation inhibitors	
NADPH oxidases inhibitors	
50 μM DPI	31±5
10mM ZnCl_2_	18±4
Peroxidases inhibitors	
0.3mM salicylic hydroxamate	27±7
10mM methimazole	35±5

Data are means ±SE of three biological replicates of 100 seeds each.

**Fig. 8.**
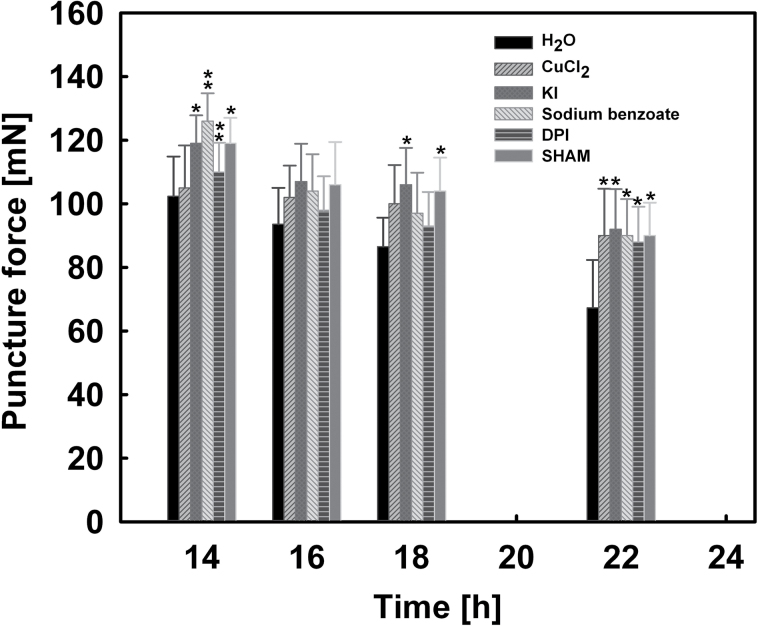
Effects of 3mM CuCl_2_, 30mM KI, 10mM sodium benzoate, 50 μM diphenylene iodonium chloride (DPI), and 0.3mM salicylic hydroxamate (SHAM) on the puncture force of CAPs of lettuce seeds. Data are means ±SE of three biological replicates of 100 seeds each. Significant differences in the data for treatments from those for water at each imbibition time point were assessed by Student’s *t*-test (*P<0.05, **P< 0.01).

**Fig. 9.**
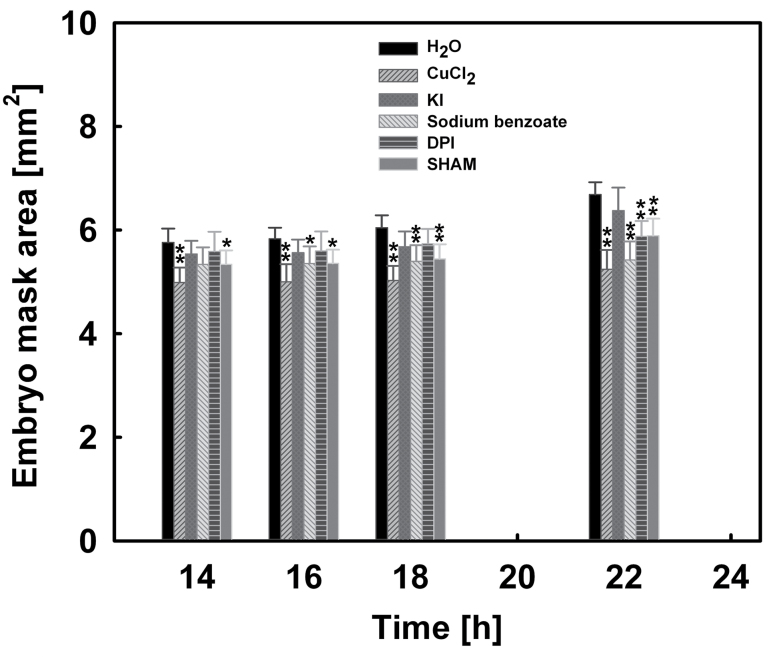
Effects of 3mM CuCl_2_, 30mM KI, 10mM sodium benzoate, 50 μM diphenylene iodonium chloride (DPI), and 0.3mM salicylic hydroxamate (SHAM) on the embryo growth potential (embryo mask area) of lettuce seeds. Data are means ±SE of three biological replicates of 100 seeds each. Significant differences in the data for treatments from those for water at each imbibition time point were assessed by Student’s *t*-test (*P<0.05, **P< 0.01).

Because the completion of seed germination is the combined result of RAD elongation growth and CAP weakening, it was then investigated whether the inhibition effect of ROS scavengers and ROS generation inhibitors on seed germination results from inhibiting the CAP weakening or the RAD elongation growth, or both. The effects of 3mM CuCl_2_, 30mM KI, 10mM sodium benzoate, 50 μM DPI, and 0.3mM salicylic hydroxamate on the puncture force of the CAP and the embryo growth potential were analysed (Figs 8, 9).

As shown in Fig. 8, all ROS scavengers and ROS generation inhibitors tested inhibited the decrease of CAP puncture force during imbibition, especially after 18h. The puncture force values of all treatments at 22h of imbibition were higher than that at 18h of imbibition in water, the threshold value for the rupture of endosperm.

In addition, treatment with 3mM CuCl_2_, 10mM sodium benzoate, 50 μM DPI, and 0.3mM salicylic hydroxamate also inhibited the increase of embryo growth potential during imbibition, especially at 18–22h (Fig. 9). However, the influence of KI (an H_2_O_2_ scavenger) on the embryo growth potential was small. This is in agreement with the observation that H_2_O_2_ was not detected by histochemical staining (Fig. 6B, D) and quantification (Fig. 6E).

### CAP rupture was increased by exogenous ROS and inducers of ROS generation

It was then investigated if inducers of ROS generation and exogenous ROS can promote seed germination. As shown in Table 2, compared with the control (water), 20 μM paraquat, 5mM H_2_O_2_, 5mM H_2_O_2_+20 μM Fe^2+^, and 5mM H_2_O_2_+20 μM Fe^2+^+1mM ascorbate increased the percentage of ruptured CAPs, but 20 μM NADPH had no effect. The effects of exogenous ROS and inducers of ROS generation on the germination of seeds were smaller than the effects of ROS scavengers and generation inhibitors. One possible explanation might be that the endogenous levels of ROS in the Guasihong lettuce seeds are probably relatively high, perhaps over a threshold value. Thus, increasing ROS levels may have an insignificant effect, while decreasing ROS levels (e.g. by ROS scavengers and generation inhibitors) below the threshold value will inhibit germination. This suggests that ROS may be necessary but not sufficient for lettuce seed germination.

**Table 2.** Effect of exogenous ROS and inducers of ROS generation on the percentage of ruptured CAP of lettuce seeds

Treatments	CAP rupture (%)
H_2_O (control)	80±7
Paraquat (20 μM)	83±5
NADPH (20 μM)	80±5
H_2_O_2_ (5mM)	88±3
H_2_O_2_ (5mM)+Fe^2+^ (20 μM)	82±5
H_2_O_2_ (5mM)+Fe^2+^ (20 μM)+ascorbate (1mM)	89±4

Data are means ±SE of three biological replicates of 100 seeds each.

The above results showed that atypical germination in lettuce seeds might be due to the inhibition of the accumulation of O_2_·^–^ and H_2_O_2_ and the activity of peroxidases in the CAP (Figs 5–7). Thus an attempt was made to rescue lettuce seeds from atypical germination by the addition of exogenous ROS and inducers of ROS generation into 0.3% SDIC. It was found that only exogenous H_2_O_2_ recovered some seeds to typical germination, in a concentration-dependent manner (Fig. 3C, D).

## Discussion

### Direct biomechanical assay of CAP weakening during lettuce seed germination

Weakening of the CAP, a prerequisite for the completion of germination, has been demonstrated by the direct measurement of puncture force in species such as tomato (Groot and Karssen, 1987) and *Lepidium sativum* (Müller *et al.*, 2006). In this approach, tomato and *L. sativum* seeds were cut into halves. The part of the seed containing the CAP (with the RAD removed) was placed in a mould, and secured in place at the CAP end. The puncturing needle was then driven through a hole in the mould.

Lettuce seeds are different from those of tomato and *L. sativum* in structure, and the measurement of endosperm puncture force has been tried previously. Nabors and Lang (1971) directly measured the force needed by the lettuce embryo to break through the endosperm by manually pulling the empty endosperm onto one end of a glass rod while the other end of the rod rested on the pan of a Mettler balance, until the rod protruded through the endosperm. However, the manipulation of pulling the endosperm onto the rod was not described, and would be difficult to carry out because the endosperm of lettuce is very thin (only two cell layers) (Jones, 1974). The method of Nabors and Lang (1971) was improved by Tao and Khan (1979), who used a circular flat-faced drill (0.2mm in diameter and attached to an Instron universal testing machine) to puncture lettuce embryos (both with and without the endosperm) placed in the centre of an aluminium block with a hole drilled through it. This method was easier than that of Nabors and Lang, but still had some disadvantages: (i) the puncture force of the endosperm was measured indirectly, by calculating the difference in measured values between naked embryo and embryo with endosperm; (ii) the endosperm was punctured from the outside instead of from the inside, thus not mimicking the RAD protrusion during germination; and (iii) the endosperm was most probably not drilled from the micropylar end, meaning that the puncture force measured was probably not that of the CAP.

In the present study, the method was improved as described in the Materials and methods using a membrane (adhesive tape) covering a hole as an endosperm holder. The method overcame the problems noted above, and is easy, objective, and accurate. For example, the puncture force values measured (67–102 mN for the CAP, 110–120 mN for the chalazal endosperm; this could explain why the endosperm ruptures at the CAP end, rather than at the chalazal end) are smaller than those reported by Tao and Khan (1979) (150–600 mN). This difference is probably due to the different method of puncturing the endosperm. In the present study the endosperm was punctured from the inner side (next to the embryo), whereas Tao and Khan (1979) punctured it from the outer side (next to the seed coat). Given that the lettuce endosperm cell walls next to the seed coat are always considerably thicker (25–30 μm) than the inner walls (6–10 μm) (Jones, 1974), it is to be expected that the puncture force of the endosperm measured from the outer side would be greater than that measured from the inner side. The present method might even be used to measure the CAP puncture force of tiny seeds such as *Arabidopsis thaliana*, a task that is currently impossible (Müller *et al.*, 2006), if a very thin needle could be inserted into the tiny CAP, for example under a microscope.

The results of this study showed that the puncture force of both intact and excised CAPs of lettuce seeds imbibed in water decreased prior to RAD protrusion, indicating that CAP weakening is an autonomous process, independent of the presence of the embryo. The fact that the puncture force of the excised CAPs was higher than that of intact endosperms implies that in intact seeds the growing embryo can also decrease the puncture force of the CAP to some extent. This supports the view proposed by Müller et al. (2013) that the endosperm cells may be weakened in relation to the opposing force of the growing RAD.

### Reason for the atypical germination of lettuce seeds in SDIC

Although the atypical germination of lettuce seeds in SDIC was reported >40 years ago, the reason for this phenomenon is unclear. SDIC is a chemical compound capable of releasing chlorine and is used to chlorinate swimming pools (Pavlišta and Haber, 1970). Thus, SDIC may damage lettuce seeds. It was shown here that SDIC specifically decreased the viability of the CAP, but not that of the lateral endosperm and the embryo. This might be due to the difference in cell wall thickness between the CAP and lateral endosperm. Given that the outer cell walls of the lateral endosperm are more than twice as thick as those of the CAP (Jones, 1974), it is suggested that: (i) the thicker outer cell walls of the lateral endosperm protect their cells from being damaged by SDIC; or (ii) the embryo enclosed inside the endosperm was protected by the endosperm. To test these hypotheses, the embryo and endosperm were isolated from water-imbibed seeds and incubated in 0.3% SDIC for different time periods before staining with TTC. The results showed that neither the embryo nor the endosperm (both the micropylar and lateral) could be stained (data not shown). This demonstrated that SDIC can penetrate through the inner cell walls of the endosperm, which are much thinner than the outer walls (Jones, 1974), and can decrease the viability of embryo when in direct contact.

Thus, the cause of the atypical germination of lettuce seeds imbibed in SDIC may be explained as follows: SDIC penetrates through the thinner outer cell walls of the CAP and decreases the viability of those cells, leading to the inhibition of its weakening. However, SDIC does not affect the viability of the embryo due to the protection of the endosperm. As a result, once the pushing force of the embryo reaches a threshold, the endosperm ruptures at a mechanically weaker region, with the unruptured CAP being pushed off intact. Supporting evidence for this view can be found in Bewley (1997), who shows that the boundary between lateral and micropylar endosperms of lettuce seeds is a narrow and a mechanically weaker region.

The phenomenon of atypical germination was also observed in *Syringa reflexa* (Juntila, 1973) and *L. sativum* (Müller *et al.*, 2009; Oracz *et al.*, 2012) where the embryo growth potential increases without endosperm weakening.

### Involvement of ROS in CAP weakening during lettuce seed germination

ROS and peroxidase activity have been shown to be associated with endosperm weakening in seeds such as *L. sativum* (Müller *et al.*, 2007, 2009; Linkies *et al.*, 2010) and tomato (Morohashi, 2002).

Here, it is shown that the amount of O_2_·^–^ and H_2_O_2_ and the activity of peroxidase in the CAP of Guasihong lettuce seeds increased during imbibition in water, and the increasing tendency coincided with the decrease of the CAP puncture force. In addition, imbibition in 0.3% SDIC inhibited the weakening process and at the same time inhibited the accumulation of O_2_·^–^ and H_2_O_2_ and the increase in peroxidase activity in the CAPs. Moreover, the addition of ROS scavengers and ROS generation inhibitors reduced the CAP weakening and resulted in a decrease in the percentage of seed germination; exogenous ROS and ROS generation promoters increased the percentage of ruptured CAPs to some extent, and the addition of H_2_O_2_ to 0.3% SDIC recovered the typical germination of some seeds.

Taken together, it is proposed that O_2_·^–^, H_2_O_2_, and peroxidase are involved in lettuce CAP weakening.

### Involvement of ROS in embryo elongation growth during lettuce seed germination

In addition to CAP weakening, RAD elongation growth is required for the completion of seed germination. Cell walls of RAD have to be loosened in order to allow cell elongation. ROS and peroxidases have been demonstrated to play a role in RAD elongation in *L. sativum* (Müller *et al.*, 2007, 2009; Linkies *et al.*, 2010). The accumulation of O_2_·^–^ in the vascular tissues is also correlated with the root elongation growth of maize seedlings (Liszkay *et al.*, 2004).

In the present study, it was found that in the RAD of Guasihong lettuce seeds imbibed in water, the amount of O_2_·^–^ and peroxidase activity increased during embryo elongation growth. SDIC at 0.3% did not inhibit embryo elongation growth or the accumulation of O_2_·^–^ and the increase in peroxidase activity in the RAD. Moreover, the addition of ROS scavengers and ROS generation inhibitors inhibited embryo elongation growth and led to a decrease in the percentage or seed germination. However, H_2_O_2_ was not detected in the RAD by histochemical staining and quantification, indicating that H_2_O_2_ has little or no role in the RAD elongation growth.

Hence, the results support that O_2_·^–^ and peroxidase play a role in embryo elongation growth in lettuce.

In conclusion, the present results suggest that ROS are involved in CAP weakening and embryo elongation growth during lettuce seed germination.

## Abbreviations:

CAP: endosperm cap
DAB: 3,3-diaminobenzidine
DPI: diphenylene iodonium chloride
NBT: nitroblue tetrazolium
RAD: radicle
ROS: reactive oxygen species
SDIC: sodium dichloroisocyanurate
TMB: 3,3′,5,5′-tetramethylbenzidine
TTC: triphenyltetrazolium chloride.
